# The Effect of a Zinc-L-Carnosine Mouthwash in the Management of Oral Surgical Wounds: Preliminary Results of a Prospective Cohort Study

**DOI:** 10.3390/dj11070181

**Published:** 2023-07-24

**Authors:** Fabio Dell’Olio, Rosaria Arianna Siciliani, Gianluigi Novielli, Angela Tempesta, Gianfranco Favia, Luisa Limongelli

**Affiliations:** Unit of Odontostomatology, Department of Interdisciplinary Medicine, University of Bari “Aldo Moro”, 70121 Bari, Italy; r.siciliani2@studenti.uniba.it (R.A.S.); gianfranco.favia@uniba.it (G.F.);

**Keywords:** zinc L-carnosine, zinc, L-carnosine, wound therapy, oral surgery, wound healing, oral mucosa, antioxidant, inflammation, split mouth

## Abstract

Background: Zinc L-carnosine promotes the transition from the inflammatory to the proliferative phase of wound healing by reducing the expression of pro-inflammatory signals and enhancing the expression of anti-inflammatory signals. This prospective cohort study aims to test the effect of a zinc–L-carnosine mouthwash in promoting oral surgical wound healing. Methods: From October 2022 to February 2023, the authors enrolled healthy adult volunteers who needed the extraction of bilateral molars at the Unit of Dentistry of the University of Bari. The authors studied the baseline wound healing of each patient after the first extraction. Three months later, the patients underwent the second extraction and rinsed their mouths with zinc–L-carnosine mouthwash twice per day for the following 28 postoperative days. For a month after each extraction, the patients received weekly follow-up visits by an oral surgeon blinded about the study to record the modified healing index score of the wounds (range 0–6 points). For statistical analysis, we used the one-tailed *t*-test for paired samples with a significance level set at *p* < 0.05 to compare the baseline scores with those recorded during the exposure to the zinc–L-carnosine mouthwash. Results: The authors enrolled four women and six men (mean age = 44.60 ± 19.22 years). On the seventh and fourteenth postoperative days, the mean difference between the modified healing index scores obtained by using the zinc–L-carnosine mouthwash and the baseline was not significant. On the twenty-first postoperative day, the mean score obtained by using the mouthwash was 5.2 ± 1.3 points and was significantly higher than the 4.7 ± 1.8 points of the baseline (*p* = 0.026). On the twenty-eighth postoperative day, the mean difference was significant as well (5.9 ± 0.3 points and 5.4 ± 1.1 points, respectively). Conclusion: The preliminary results of this study showed that the zinc–L-carnosine mouthwash improved the quality of oral surgical wound healing.

## 1. Introduction

Surgical oral wound healing is a complex interplay involving keratinocytes, fibroblasts, endothelial cells, immune cells, and extracellular matrix (ECM) [1,2]. During wound healing, four sequential and overlapping phases occur: hemostasis, inflammation, proliferation, and remodeling [1,2]. The persistence of inflammation impairs wound healing, thus granulomas, fistulas, dehiscence, ulcers, and excessive bleeding occur [3]. To avoid such postoperative complications, the literature suggests several treatment approaches improving oral wound healing; the current therapies employ a delivery vehicle to set up a local environment promoting the healing process [3]. Scaffolds, gels, and topical ointments are the most common solutions for oral wound therapy, whereas low-intensity pulsed ultrasounds and low-level laser therapy (LLLT) are emerging alternative approaches [3,4]. Among topical ointments, molecules such as hyaluronic acid promote wound healing by providing constituents of the ECM, whereas others, such as anthocyanin, facilitate wound healing by providing local anti-inflammatory and antioxidant effects [3,5]. Zinc L-carnosine (ZnC) is an antioxidant compound composed of an L-carnosine molecule chelating a zinc atom [6]. Zinc is an essential mineral acting as a cofactor of many enzymatic pathways, which belong to the cellular repair, cell proliferation, and healing of epithelial wounds, whereas L-carnosine (β-alanyl-L-histidine) is a metal-chelating dipeptide showing functions such as antioxidant, scavenger, buffer, superoxide dismutase-like, and protective for wounds [6,7]. During wound healing, ZnC promotes the transition from the inflammatory to the proliferative phase by reducing the expression of pro-inflammatory signals and enhancing the production of anti-inflammatory cytokines [7,8]; therefore, ZnC may be useful in oral wound therapy. In addition, ZnC formulations prolong the interaction with the wound by using sodium alginate, which adheres to the mucosa and provides a physical barrier against local trauma [9]. The current prospective cohort study aims to test the effect of a ZnC mouthwash in promoting oral surgical wound healing.

## 2. Materials and Methods

From October 2022 to February 2023, the authors performed the current prospective cohort study at the Unit of Dentistry of the “Aldo Moro” University of Bari, Italy. The study complied with the Declaration of Helsinki and received the approval of the local Ethical Committee (code 7373/CE; approved on 21 September 2022). Before joining the study, all participants provided written informed consent. The authors enrolled volunteers according to the following inclusion criteria: age of at least 18 years; the need for the extraction of bilateral molars; and a medical history without comorbidities and medications interfering with wound healing. Therefore, diabetes, chronic inflammatory diseases, immunodepression, radiation therapy in the head and neck region, immunosuppressors, corticosteroids, chemotherapy, angiogenesis inhibitors, and bisphosphonates were the exclusion criteria of the study. The authors studied a mouthwash containing 39.53 mg of ZnC per 10 mL of suspension based on purified water and sodium alginate, together with magnesium hydroxide, glycerin, sucralose, mint essential oil, and methyl-para-hydroxybenzoate (Hepilor Collutorio^®^, Azienda Farmaceutica Italiana S.r.l., Sant’Egidio alla Vibrata, Italy). The authors used the modified Landry’s healing index (MHI) by Pippi et al. [10] as the main outcome of the study because this index allowed the assessment of the quality of the secondary intention healing of soft tissues associated with post-extractive dental sockets (Table 1). The MHI score ranges between 0 points and 6 points, meaning the lowest and highest quality of healing, respectively.

The authors calculated the sample size to establish the participants needed to see a relevant improvement in a paired design. The authors imputed a one-tailed 0.05 significance level, 80% power, an expected mean difference of 1 point of MHI, and an acceptable standard deviation of 0.5 points into the statistical software. The esteemed sample size was 4 patients; however, the authors decided to include all patients enrolled during the study period. For a month after each extraction, the patients received weekly follow-up visits by an oral surgeon blinded to the study to avoid measurement bias. The oral surgeon recorded the MHI score and photographed the wounds with a 25 MP camera. In addition, the authors assessed wound healing by calculating the percentage healing index (PHI) [11]. According to the calculation of PHI, the authors used graphics software (Adobe Photoshop CC™, Adobe Systems, San Jose, CA, USA) to delimit and measure the area of unhealed wounds. Such an index required an intraoral benchmark to calibrate the measures in the pictures; however, the authors solved such an issue by calculating a calibration factor based on the area of the occlusal surface of an adjacent tooth to the extraction site:

$$CalibrationFactor=\frac{\left(areaofthereferencetooth\in thepicturetakenafterXpostoperativedays\right)}{\left(areaofthereferencetooth\in thepicturetakenaftersurgery\right)}$$

Then, the authors multiplied each area for the respective calibration factor; eventually, the authors calculated the PHI for each wound and time-point with the following formula [11]:

PHI=[1−((Area measured after X postoperative days)⁄(Area measured after surgery)]∗100)

The authors calculated the PHI of the healed wounds by measuring the area of the scars; therefore, the smaller the scars, the greater the PHI. After the first extraction, the patients cleaned their wounds by using saline solution twice per day, and the authors studied the quality of the wound healing of the participants in baseline conditions. Three months later, each patient underwent the extraction of the contralateral molar by the same oral surgeon. After the second extraction, the patients cleaned their wounds by using the saline solution and rinsed with 10 mL of the ZnC mouthwash twice per day for the following 28 postoperative days (79.06 mg of ZnC daily), and the authors studied the quality of the wound healing conditioned by ZnC. The authors conducted the statistical analysis by using IBM SPSS Statistics for Windows, version 27.0. For each postoperative week, the authors performed the one-tailed *t*-test for paired samples with a significance level set at *p* < 0.05 to compare the baseline MHI scores with those conditioned by the ZnC mouthwash. The null hypothesis (H_0_) meant that the mean difference of the MHI scores between baseline and ZnC was 0 and that the mouthwash did not affect the quality of wound healing. The alternative hypothesis (H_1_) meant that the mean difference was higher than 0 and that the mouthwash improved the quality of wound healing. In addition, the authors calculated the frequency of completely healed wounds in baseline and ZnC. Then, the authors performed the same analysis for PHI by conducting the Wilcoxon test for the non-parametric comparison of paired groups. The null hypothesis (H0) meant that the difference in median PHI between baseline and ZnC was 0 and that the mouthwash did not affect the quality of wound healing. The alternative hypothesis (H1) meant that the difference in median PHI was higher than 0 and that the mouthwash improved the quality of wound healing.

## 3. Results

From October 2022 to February 2023, ten patients completed the study protocol, four women and six men with a mean age of 44.60 ± 19.22 years (Table 2). The authors did not record intraoperative and postoperative complications. The patients did not experience adverse reactions to the ZnC mouthwash. In addition, no patients withdrew from the study or were lost in follow-up. 

On the seventh and fourteenth postoperative days, the mean difference between the baseline MHI scores and those obtained by using the ZnC mouthwash was not significant (*p* = 0.172 and *p* = 0.084, respectively; Table 3). On the twenty-first postoperative day, 5.2 ± 1.3 points was the mean score obtained by using the mouthwash and was significantly higher than the 4.7 ± 1.8 points of the baseline (*p* = 0.026). On the twenty-eighth postoperative day, the mean difference was significant as well (5.9 ± 0.3 points and 5.4 ± 1.1 points, respectively; *p* = 0.048). 

On the twenty-first postoperative day, three wounds completed the healing in baseline against the six in ZnC. In addition, the healed wounds were six in baseline and nine in ZnC on the twenty-eighth postoperative day. Table 4 shows the findings regarding the PHI by reporting the median and the interquartile range for each time-point. On the seventh and fourteenth postoperative days, the difference between the median of the baseline PHI and those obtained by using ZnC was not significant (*p* = 0.189 and *p* = 0.090, respectively). On the twenty-first postoperative day, the median PHI obtained by using the mouthwash was 85.5% and was significantly higher than the 80.4% of the baseline (*p* = 0.29). On the twenty-eighth postoperative day, the difference was significant as well (95.1% and 87.9%, respectively; *p* = 0.007).

**Figure 1:**
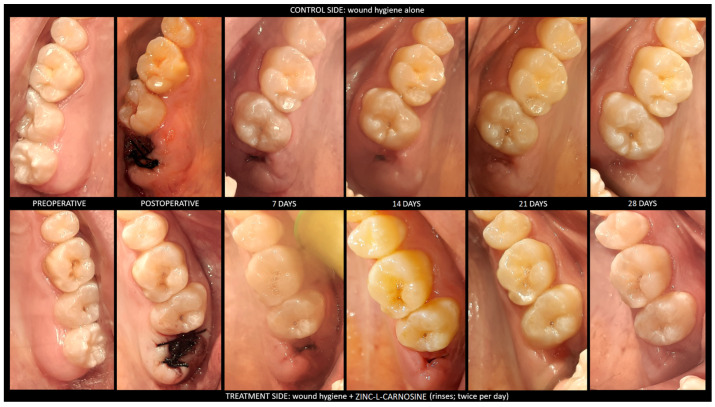
Clinical case. Figure 1 shows a clinical case to provide an example of the assessment and recording of the healing index. On the seventh postoperative day, both wounds scored 2 points because they showed no signs of bleeding and suppuration. On the fourteenth postoperative day, both wounds had a healing score of 3 points because the gingiva turned pink in both cases. On the twenty-first postoperative day, only the wound exposed to ZnC showed no signs of swelling, and then the healing scores of the baseline and ZnC were 4 points and 3 points, respectively. On the twenty-eighth postoperative day, the wound that received ZnC completed the closure of the margins with epithelium, whereas the baseline wound healing showed red exposed connective tissue; the healing scores were 6 points and 4 points, respectively. The complete healing of the baseline wound occurred outside of the observational period after the fourth postoperative week.

**Figure 2:**
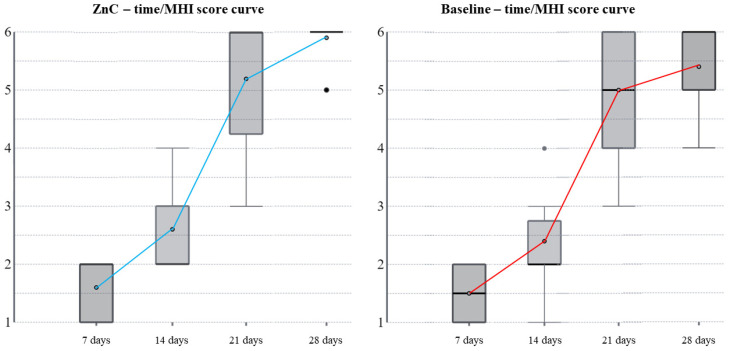
Variation of MHI scores over time in baseline conditions and by using the ZnC mouthwash. Figure 2 shows the MHI score growing slowly during the first and second postoperative weeks. Then, the score grows quickly during the third postoperative week; eventually, the MHI score grows slowly again during the fourth postoperative week. Such a pattern occurs both in baseline conditions and by using the ZnC mouthwash; in addition, the growth of the MHI score is faster by using the ZnC mouthwash than baseline during the third and the fourth postoperative weeks. Abbreviations: MHI, modified healing index; ZnC, zinc–L-carnosine.

**Figure 3:**
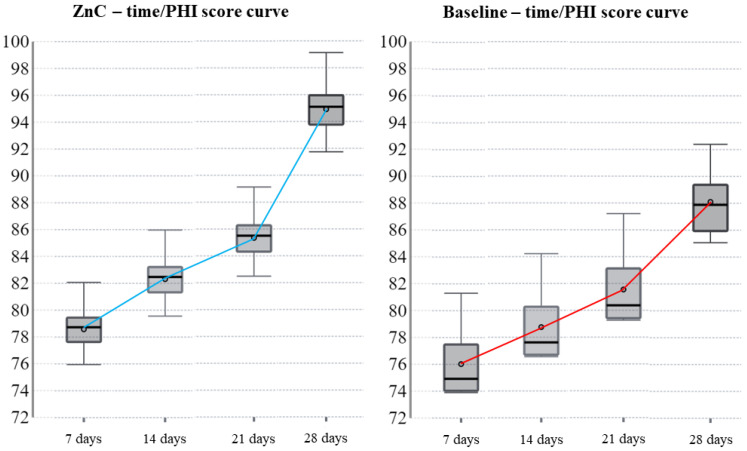
Variation of PHI scores over time in baseline conditions and by using the ZnC mouthwash. Figure 3 shows the PHI score growing slowly during three postoperative weeks; then, the score grows quickly during the fourth postoperative week. Such a pattern occurs both in baseline conditions and by using the ZnC mouthwash; overall, the growth of the PHI score is faster by using the ZnC mouthwash than baseline for all the healing and reaches the maximum during the fourth postoperative week.

In the current study results, the patients showed an improved quality of secondary intention healing of the soft tissues associated with post-extractive dental sockets after rinsing with 79.08 mg of ZnC per day. In addition, the ZnC mouthwash induced faster healing with smaller scars than the baseline. Figure 1 shows a clinical case in detail, and Figure 2 depicts the variation of MHI scores over time both in baseline conditions and by using the ZnC mouthwash. Figure 3 shows the growth of PHI over time in baseline and ZnC.

## 4. Discussion

Oral wound healing is a complex interplay involving keratinocytes, fibroblasts, endothelial cells, recruited immune cells, and the ECM [3]. The several sequential and overlapping events occurring during wound healing are commonly divided into four phases: hemostasis, inflammatory, proliferative, and remodeling [1,2]. Immediately after the injury, the hemostasis phase starts, and the exposition of sub-endothelium, collagen, and tissue factor (TF) triggers the aggregation and degranulation of platelets, thus allowing the formation of the blood clot and the releasing of several chemokines and growth factors [2]. The degranulation of platelets activates the complement cascade, which contributes to both the start of inflammation and the killing of bacteria in the wound site [8]. The inflammatory phase begins five or six hours after surgery and ends between the first and the third postoperative days or between the third and the fifth postoperative days [1,2,8]. Inflammation is responsible for swelling, redness, heat, and pain in the surgical site [1]. During the inflammatory phase, pro-inflammatory cytokines, such as tumor necrosis factor α (TNF-α), interleukin-1β (IL-1β), and IL-6, recruit peripheral leucocytes in the wound [12]. Early recruited neutrophils cleanse both debris and invading pathogens in the wound site while amplifying the inflammation by releasing the same pro-inflammatory cytokines described above; later, macrophages migrate for removing pathogens, clearing apoptotic neutrophils, and thus achieving a good environment for the healing process [2,3,12]. If the wound does not become infected, neutrophils abandon the site between two and five days [8]; when neutrophils remain in the wound site over time, their proteases degrade ECM and the release of reactive oxygen species (ROS), causing oxidative stress damage to the tissues, thus delaying the healing [8]. Macrophages occur in the wound site approximately three days after the injury and are involved in the regulation of scar formation and wound closure [3,8,12]. During the healing, macrophages are present both in M1 and M2 forms [12]. The M1 form is the pro-inflammatory, activated phenotype responsible for phagocytosis and the further production of inflammatory mediators [8]; the M2 form is the anti-inflammatory phenotype and has functions related to tissue repair [12]. Thus, the balance between pro-inflammatory and anti-inflammatory signals is important during this phase [2]. A long-lasting inflammatory phase damages the tissues during healing; in addition, chronic inflammation dysregulates the differentiation of keratinocytes and induces pathological healing [2,8]. The proliferative phase starts on the third postoperative day and lasts for up to three weeks [1,8]; during this phase, the granulation tissue replaces the blood clot and leads to the formation of a mature scar [1,2]. The granulation tissue contains inflammatory cells, local and bloodborne fibroblasts, and new blood vessels in a loose collagenous ECM [2,3]. Myofibroblasts differentiate in the wound as a response to the mechanical tension increase, and aid wound contraction [2,3]. The epithelium repairs from the cut wound edges to the new fibrin-rich matrix [3]. Transforming growth factor β1 (TGF-β1), TGF-β2, fibroblast growth factor (FGF), epidermal growth factor (EGF) [3], IL-6, IL-8, and vascular endothelial growth factor (VEGF) are involved in this phase [2,3]. Angiogenesis and the endothelial cell sprouting driven by VEGF have pivotal importance for the success of healing [3,5]. The dysregulation of the proliferative phase causes scar contracture and an impaired function of the surgical site [1]. The remodeling phase starts during the twenty-first postoperative day and can last from a few months to several years [1,2,8]; during this period, fibroblasts produce type I collagen, replacing the type III collagen in the ECM of the scar [1]. The remodeling phase requires a balance between apoptosis and proliferation [2]; when the dysregulation of the remodeling occurs, the wound develops an exuberant scar if the speed of proteolytic activity is lower than the speed of type I collagen production [1], whereas the wound dehiscence occurs if the proteolysis is quicker than the collagen deposition [1]. Furthermore, the quality of wound closure depends on the dimensions of the loss of substance, and local and systemic factors [1] such as hypoxia, infections, tumors, metabolic disorders (e.g., diabetes mellitus), debris, necrotic tissues, and a diet lacking in proteins, vitamins and/or minerals, which may impair surgical wound closure [5]. Nevertheless, the meticulous tissue handling, suturing, and prevention of infections are mandatory [2]. Postoperative wound management aims to achieve healing without pain, discomfort, and scarring as soon as possible. Current oral wound treatment approaches set up a local environment, promoting healing [3]. The main functions of wound healing therapies are the prevention of infections, mechanical protection, the administration of nutritional support, the delivery of the ECM’s constituents, and both anti-inflammatory and antioxidant effects [3,4,5,8]. Scaffolds, gels, and topical ointments are the most common solutions for oral wound therapy, whereas low-intensity pulsed ultrasounds and LLLT are emerging alternative approaches [3,4]. In addition, experimental wound healing therapies comprehend the delivery of fibroblasts and VEGF, immunomodulation by FTY720, or the application of non-steroidal anti-inflammatory drugs [3]. The composition of the scaffolds makes them classifiable into biological, synthetic polymeric, and hybrid biological–synthetic polymer ones [3]. Biological scaffolds are vehicles for cells and growth factors, such as keratinocytes, fibroblasts, and bFGF [3]. Polymeric scaffolds deliver drugs, such as glutathione, FTY720, and curcumin [3]. The low-intensity pulsed ultrasounds promote the healing of bone fractures and soft tissues by inducing the release of bFGF and TGF-β [3]. LLLT promotes oral wound healing by increasing cellular metabolism, accelerating keratinocyte migration, and reducing inflammation [4]. Topical ointments and gels promote wound healing in multiple ways, such as providing constituents of the ECM (e.g., hyaluronic acid) and exerting local anti-inflammatory and antioxidant (e.g., anthocyanin) [3,5]. In the current study, the authors studied the application of a ZnC suspension in sodium alginate mouthwash as a wound-healing agent in oral surgery. Oral surgical wounds heal according to two main clinical patterns: primary intention and secondary intention [10]. At the end of the surgery, the primary intention healing occurs because the lining tissues become approximated by covering all the underlying tissues; the primary intention is the fastest kind of healing and carries a low risk of infection and scarring [10]. The secondary intention healing occurs because the lining tissues do not become approximated, such as in extraction sockets, apically repositioned flaps, or a full-thickness loss of substance [10]. After the flapless extraction of a tooth, the blood clot fills the socket within a few minutes and granulation tissue replaces the blood clot within seven days; such a process allows for the completion of re-epithelization [10]. Mucosa covers the socket completely within a period of up to five weeks [10]. After the end of the healing of soft tissues, the surface of the socket maintains a concave shape until the bone heals as well [10]. Secondary intention healing occurs with the risk of several postoperative complications [3,10]. Even open surgery is not free from the complications of other extractions; therefore, wound therapy is also advantageous in this kind of dental extraction [3,8,10]. The purpose of the ZnC mouthwash is the promotion of postoperative oral surgical wound healing by using the anti-inflammatory and antioxidant effects of such a molecule, as other wound healing agents possess [3,6,13]. Before using such a mouthwash in patients with impaired wound healing or pathological wounds, the authors experimented with ZnC in healthy patients with an ideal repair process. Therefore, the authors enrolled patients showing medical histories without comorbidities and medications interfering with wound healing; in addition, the authors excluded patients carrying diabetes, chronic inflammatory diseases, immunodepression, radiation therapy in the head and neck region, immunosuppressors, corticosteroids, chemotherapy, angiogenesis inhibitors, and bisphosphonates. After removing the confounding factors related to the systemic conditions and achieving good oral hygiene, the healing differences between baseline and mouthwash showed the effects of the ZnC; similar studies employed such a design to experiment with other types of wound therapy in oral surgery [1]. In the current study, the ZnC complied with the aims of the healing agents because the wounds completed the secondary intention healing in most of the patients after the administration of the ZnC mouthwash between the third and the fourth postoperative weeks, whereas the wounds completed the secondary intention healing in baseline conditions in most of the patients after the fourth postoperative week. During the postoperative period, 50% of patient experience bleeding, infections, dehiscence, or adverse tissue changes as complications; in addition, the highest incidence of complications occurs in anterior segments and bone surgery [10]. The authors also complied with a washout period of three months between the two extractions to prevent any improvement in the outcome of the second extraction that could have been induced by the first one. The wound healing scales are useful for the postoperative monitoring of wound healing, which is a concern for oral surgeons [1,10]; however, the use of such scales is still infrequent in oral surgery [1]. In the current study, the authors used the modified Landry’s healing index by Pippi et al. [10] to assess the secondary intention healing of soft tissues surrounding post-extractive dental sockets. Such an index provides a detailed assessment of the early phases of the healing of soft tissues associated with dental sockets [1] and has been used successfully in other clinical studies [10]. However, the MHI requires a clinical observer who could potentially bias the measurement through a subjective point of view. Therefore, the authors also used PHI as an objective index to assess wound healing [11]. ZnC is a compound based on zinc chelated with L-carnosine [6]. Zinc is an essential mineral and a cofactor of many enzymatic pathways involved in cell proliferation, cell repair, and the healing of epithelial tissue wounds [6]. On the contrary, zinc deficiency leads to growth retardation, taste disorders, and epithelial symptoms [6]. In addition, zinc is both an antioxidant and a signal for the expression of other antioxidants [7]. L-carnosine is a metal-chelating dipeptide showing functions such as antioxidant, scavenger, buffer, superoxide dismutase-like, and protective for wound healing [6,7]. Furthermore, L-carnosine enhances the absorption and release of zinc in target tissues [6]. Many authors conducted studies about the properties of ZnC in animal models, such as murine gastric mucosa [6,13]. ZnC mainly showed an anti-inflammatory effect, antioxidant action [6], and the ability to increase other antioxidant activities, such as superoxide dismutase (SOD)-like and glutathione-S-transferase (GST) [7]. Ko et al. studied ZnC administration on acetic acid-induced gastric ulcers in rats and found both the overexpression of pro-proliferation signals and downregulation of pro-inflammatory signals [6]. ZnC induced the expression of bFGF, VEGF, and ornithine decarboxylase; in addition, ZnC inhibited the production of TNF-α, IL-1β, and macrophage inflammatory protein 2 (MIP-2) [6]. ZnC reduced dose-dependently pro-inflammatory cytokines such as IL-1β, IL-8, IL-6, and TNF-α in rats affected by gastric ulcers [6]; in such studies, the rats which received ZnC showed concentrations of antioxidants such as SOD-1, SOD-2, heme oxygenase-1 (HO-1), peroxiredoxin-1 (Prx-1), and Prx-5 higher than controls [6]. In addition, ZnC causes a significant increase in platelets-derived growth factor (PDGF), nerve growth factor (NGF), and VEGF [6]. For six hours, ZnC supplementation inhibited the nuclear factor kappa-light-chain-enhancer of activated B cells (NF-kB) in murine models [7]; other studies found that the ZnC-induced inhibition of NF-kB is dose-dependent in rats with inflammatory states provoked by lipopolysaccharide (LPS) [14]. NF-kB is a transcriptional factor that induces the expression of several pro-inflammatory genes [7]. NF-kB is low during the early days of wound healing; in contrast, the activity of such a transcription factor grows in the late phases of the healing process [4]. During such phases, the inhibition of the NF-kB has a role in promoting healing; for example, LLLT is a wound treatment approach that also uses the inhibition of NF-kB to promote healing [4]. ZnC reduced the expression of the inducible nitric oxide synthase (iNOS) in rats stimulated by LPS [13,15]. In addition, ZnC reverted lipid peroxidation and neutrophil accumulation induced by aspirin in rat gastric mucosa [9]. The Western blot analysis showed a dose-dependent expression of cytoprotective compounds, such as heat shock protein 25 kDa (HSP-25), HSP-27, and HSP-72 in the gastric and colonic mucosa of rats exposed to ZnC [7,9]. Several conditions that can damage the cells, such as heat shock, oxidative stress, and heavy metals, induce the expression of HSPs [7]; in addition, the family of HSP70 regulates the expression of inflammatory genes and inhibits the activation of NK-kB, therefore exhibiting a higher anti-inflammatory effect than other HSPs [7,16]. ZnC inhibited myeloperoxidase (MPO) and, in addition, reduced TNF-α and interferon γ (INF-γ) in injured murine colonic mucosa [7]. Gao et al. induced a significant bone regeneration in the cranium of osteoporotic rats by implanting ZnC-loaded polycaprolactone/gelatin membranes [17], whereas Ko et al. accelerated bone callus conversion to mature bone in mice with femoral fractures by orally administering 25 mg/Kg of ZnC per day [18]. Further evidence about the properties of ZnC derives from in vitro studies [6]. Other experiments demonstrated that ZnC inhibited the production of superoxide and hydroxyl radicals in polymorphonuclear leukocytes [7]. ZnC showed a dose-dependent pro-migratory effect and stimulated proliferation in human epithelial cells during wound healing [6]. ZnC prevented DNA and chromosomal damage and maintained zinc concentrations between 4 μmol and 16 μmol in cultured human oral keratinocytes [6]. Eventually, studies found that ZnC protected patients from oxidative stress, the promotion of osteogenesis, and the differentiation of osteoclasts in models using cultures of mouse bone marrow-derived monocytes, MC3T3-E1 murine cells, and human bone marrow-derived mesenchymal stem cells, thus suggesting a role of ZnC in bone remodeling [17,18]. Several pre-clinical and clinical studies report the safety of ZnC, and such a supplementation is common in the USA to stimulate the repair of gastric mucosa in patients with peptic ulcers [6,13]. The administration of ZnC improved symptoms and clinical signs of gastric ulcers (50 mg–150 mg; twice per day) [6]. Tan et al. studied the supplementation of ZnC (75 mg or 150 mg, twice per day) as an adjuvant for the eradication of *Helicobacter pylori* in adults receiving triple therapy (omeprazole 20 mg, amoxicillin 1 gr, and clarithromycin 500 mg), and the authors found that patients who received ZnC had a significantly superior eradication rate than those who received triple therapy alone [19]. There is evidence that ZnC enhances mucosal repair also in other parts of the gastrointestinal tract, such as oral mucosa, because ZnC targets all epithelial tissues [6]. Many studies support the administration of ZnC to manage taste disorders and oral mucositis caused by chemotherapy and radiotherapy [6]. Oral mucositis induces oral erythema, ulcers, and the overall impairment of the quality of life and occurs as a complication in 75% of head and neck radiotherapy and chemotherapy [20]. Patients with oral mucositis show severe pain, odynophagia, dysgeusia, malnutrition, dehydration, prolonged hospitalization, and an early disruption of the therapy [6,9]. The incidence of oral mucositis is three times higher in pediatric and adolescent patients than in adults because of the higher proliferation rate of basal cells [20]. The initiation phase of oral mucositis starts with the damage by reactive oxygen species; then, NF-kB, TNF-α, IL-1β, and IL-6 carry the inflammation phase; immune cells cause the apoptosis of the epithelium and lead to the ulceration phase; eventually, the healing phase occurs [9,20]. During the ulceration phase, oral mucosa is susceptible to secondary infection, especially because of the myelosuppression secondary to chemotherapy and/or radiotherapy [9,13]. Hayashi et al. administered P-AG to patients with hematological malignancy who underwent high-dose chemotherapy, radiotherapy, and following hematopoietic stem cell transplantation (HSCT) [9]. The incidence of grade 3 oral mucositis was lower in patients who received P-AG than in controls (0% against 45%), and the pain was significantly lower in the P-AG group, thus resulting in a smaller use of local anesthetics as analgesics than in controls [9]. Later, Hayashi et al. developed an orally applicable 18.75 mg P-AG tablet and tested its efficacy against the P-AG suspension in patients with hematological malignancies [9]. Patients who received P-AG tablets developed grade 3 oral mucositis in 6.3% of cases, whereas those who received P-AG suspension developed it in 3% [9]. Suzuki et al. analyzed the medical records of 104 patients who experienced oral mucositis during radiotherapy for head and neck cancer, and 79 of them received P-AG as a two-minute rinse followed by swallowing four times per day [21]. Grade 3 oral mucositis occurred in 16.5% of patients who received P-AG against 52% of controls [21]. In addition, Suzuki et al. observed that the median duration of radiotherapy and the median time to discharge after the completion of radiotherapy were significantly lower in the P-AG group than in controls [21]. Funato et al. administered P-AG in children undergoing high-dose chemotherapy, radiotherapy, and following HSCT due to hematological malignancies [20]. Grade 3 oral mucositis occurred in 20% of children who received P-AG against 33.3% of controls [20]. In addition, all controls needed opioids against the 30% of children who received P-AG [20]. Eventually, Tsubura-Okubo et al. observed the reduction in pain, taste disorders, and incidence of oral mucositis by rinsing and swallowing ZnC in polyacrylic acid solution (PPAA) in patients that received chemotherapy and HSCT [22]. In addition, the severity of acute graft-versus-host disease (GVHD) was significantly lower in patients that received PPAA than in controls [22]. Taste disorders are another intraoral clinical application of ZnC because zinc is a cofactor of taste buds’ enzymes [6]. The daily supplementation of 150–300 mg of ZnC improved the symptoms of patients with taste disorders not associated with chemoradiotherapy [6]. Dysgeusia occurs in 41.8–76% of patients who receive chemotherapy, most likely because chemotherapeutic agents inhibit the renewal of taste buds and cause zinc deficit [23]. Fujii et al. studied 40 patients who developed severe taste disorders due to chemotherapy and who received a supplementation of 150 mg of ZnC twice per day [23]. Patients that received ZnC supplementation recovered from taste disorders in a shorter time than controls [23]. This study carries the limits of observational studies and the small number of participants; in addition, the study lacks histological examination to assess the quality of healed gingiva because of the ethical concerns related to performing biopsies for research purposes only. However, the study provides a proof of concept: ZnC can be employed in oral surgery as an adjuvant to the secondary intention healing of the mucosa surrounding the dental sockets; the results are preliminary, but are interesting for both clinicians and researchers. Several studies showed the dose-dependent response of tissues to ZnC; therefore, the following research should focus on testing the effects of more than two administrations of the ZnC mouthwash. In addition, the current study calculated the sample size for conducting an RCT, which is another branch to develop research on the oral surgical applications of ZnC. Dividing the study population into two and administering the mouthwash to only half of it after the first extraction, and then to the other half after the second extraction, would be a choice of design to avoid biases in further studies. In addition, the design could improve by supplementing further rigorous tests to study the effects of ZnC, such as the microbiological assessment of the wounds. Eventually, a further development of the current research could test the application of ZnC for the postoperative wound healing of other branches of oral surgery, such as implantology, biopsies, and laser surgery. 

## 5. Conclusions

The preliminary results of this study showed that the ZnC mouthwash improved the quality of oral surgical wound healing. In addition, the ZnC mouthwash induced faster healing with smaller scars than the baseline. After the completion of the current study, the following steps of the research will check those results in an RCT design and test the ZnC in branches such as oral laser surgery.

## Figures and Tables

**Table 1:** Modified healing index by Pippi R. et al. [10].

Item	0 Points	1 Point
Color of gingiva	Red	Pink
Granulation tissue	Present	Absent
Re-epithelization	Exposed connective tissue	Complete re-epithelization
Swelling	Present	Absent
Bleeding on palpation	Present	Absent
Suppuration	Present	Absent

**Table 2:** Characteristics of the study patients.

Characteristics	Data
n	10
Male/Female	6/4
Mean Age and Standard Deviation	44.60 ± 19.22 years
Lost in Follow-Up	0
Withdrawals	0
Intraoperative Complications	0
Postoperative Complications	0
Adverse Reactions to the HEPILOR^®^ Collutorio	0

**Table 3:** Modified healing index: summary of findings.

Time-Points	Baseline	Zinc–L-Carnosine	Mean Difference	*p*-Value
7 days	1.5 ± 0.5	1.6 ± 0.5	0.1 ± 0.1	*0.172*
14 days	2.4 ± 1.0	2.6 ± 0.8	0.2 ± 0.1	*0.084*
21 days	4.7 ± 1.8	5.2 ± 1.3	0.5 ± 0.2	*0.026*
28 days	5.4 ± 1.1	5.9 ± 0.3	0.5 ± 0.2	*0.048*

**Table 4:** Percentage healing index: summary of findings.

Time-Points	Baseline	Zinc–L-Carnosine	*p*-Value
7 days	74.9% (3.4%)	78.7% (1.8%)	*0.189*
14 days	77.6% (3.6%)	82.5% (1.9%)	*0.090*
21 days	80.4% (3.7%)	85.5% (2.0%)	*0.029*
28 days	87.9% (3.4%)	95.1% (2.2%)	*0.007*

## Data Availability

The data presented in this study are available on request from the corresponding author. The data are not publicly available due to privacy concerns.

## References

[1] Hamzani Y., Chaushu G. (2018). Evaluation of Early Wound Healing Scales/Indexes in Oral Surgery: A Literature Review. Clin. Implant Dent. Relat. Res..

[2] Wang P.-H., Huang B.-S., Horng H.-C., Yeh C.-C., Chen Y.-J. (2018). Wound Healing. J. Chin. Med. Assoc..

[3] Toma A.I., Fuller J.M., Willett N.J., Goudy S.L. (2021). Oral Wound Healing Models and Emerging Regenerative Therapies. Transl. Res. J. Lab. Clin. Med..

[4] de Farias Gabriel A., Wagner V.P., Correa C., Webber L.P., Pilar E.F.S., Curra M., Carrard V.C., Martins M.A.T., Martins M.D. (2019). Photobiomodulation Therapy Modulates Epigenetic Events and NF-ΚB Expression in Oral Epithelial Wound Healing. Lasers Med. Sci..

[5] Bonifacio M.A., Cassano A., Vincenti A., Vinella A., Dell’Olio F., Favia G., Mariggiò M.A. (2023). In Vitro Evaluation of the Effects of Hyaluronic Acid and an Aminoacidic Pool on Human Osteoblasts. Biomedicines.

[6] Hewlings S., Kalman D. (2020). A Review of Zinc-L-Carnosine and Its Positive Effects on Oral Mucositis, Taste Disorders, and Gastrointestinal Disorders. Nutrients.

[7] Ooi T.C., Chan K.M., Sharif R. (2017). Antioxidant, Anti-Inflammatory, and Genomic Stability Enhancement Effects of Zinc l-Carnosine: A Potential Cancer Chemopreventive Agent?. Nutr. Cancer.

[8] Landén N.X., Li D., Ståhle M. (2016). Transition from Inflammation to Proliferation: A Critical Step during Wound Healing. Cell. Mol. Life Sci..

[9] Hayashi H., Kobayashi R., Suzuki A., Yamada Y., Ishida M., Shakui T., Kitagawa J., Hayashi H., Sugiyama T., Takeuchi H. (2016). Preparation and Clinical Evaluation of a Novel Lozenge Containing Polaprezinc, a Zinc-L-Carnosine, for Prevention of Oral Mucositis in Patients with Hematological Cancer Who Received High-Dose Chemotherapy. Med. Oncol. Northwood Lond. Engl..

[10] Pippi R. (2017). Post-Surgical Clinical Monitoring of Soft Tissue Wound Healing in Periodontal and Implant Surgery. Int. J. Med. Sci..

[11] Palaia G., Tenore G., Tribolati L., Russo C., Gaimari G., Del Vecchio A., Romeo U. (2019). Evaluation of Wound Healing and Postoperative Pain after Oral Mucosa Laser Biopsy with the Aid of Compound with Chlorhexidine and Sodium Hyaluronate: A Randomized Double Blind Clinical Trial. Clin. Oral Investig..

[12] Kotwal G.J., Chien S. (2017). Macrophage Differentiation in Normal and Accelerated Wound Healing. Results Probl. Cell Differ..

[13] Li M., Sun Z., Zhang H., Liu Z. (2021). Recent Advances on Polaprezinc for Medical Use (Review). Exp. Ther. Med..

[14] Ooi T.C., Chan K.M., Sharif R. (2016). Zinc Carnosine Inhibits Lipopolysaccharide-Induced Inflammatory Mediators by Suppressing NF-Κb Activation in Raw 264.7 Macrophages, Independent of the MAPKs Signaling Pathway. Biol. Trace Elem. Res..

[15] Xie W., An L., Liu Z., Wang X., Fu X., Ma J. (2023). Therapeutic Effect of Polaprezinc on Reflux Esophagitis in the Rat Model. Dig. Dis. Sci..

[16] Relja B., Land W.G. (2020). Damage-Associated Molecular Patterns in Trauma. Eur. J. Trauma Emerg. Surg. Off. Publ. Eur. Trauma Soc..

[17] Gao X., Al-Baadani M.A., Wu M., Tong N., Shen X., Ding X., Liu J. (2022). Study on the Local Anti-Osteoporosis Effect of Polaprezinc-Loaded Antioxidant Electrospun Membrane. Int. J. Nanomed..

[18] Ko E.A., Park Y.J., Yoon D.S., Lee K.-M., Kim J., Jung S., Lee J.W., Park K.H. (2022). Drug Repositioning of Polaprezinc for Bone Fracture Healing. Commun. Biol..

[19] Tan B., Luo H.-Q., Xu H., Lv N.-H., Shi R.-H., Luo H.-S., Li J.-S., Ren J.-L., Zou Y.-Y., Li Y.-Q. (2017). Polaprezinc Combined with Clarithromycin-Based Triple Therapy for Helicobacter Pylori-Associated Gastritis: A Prospective, Multicenter, Randomized Clinical Trial. PLoS ONE.

[20] Funato M., Ozeki M., Suzuki A., Ishihara M., Kobayashi R., Nozawa A., Yasue S., Endo-Ohnishi S., Fukao T., Itoh Y. (2018). Prophylactic Effect of Polaprezinc, a Zinc-L-Carnosine, Against Chemotherapy-Induced Oral Mucositis in Pediatric Patients Undergoing Autologous Stem Cell Transplantation. Anticancer Res..

[21] Suzuki A., Kobayashi R., Shakui T., Kubota Y., Fukita M., Kuze B., Aoki M., Sugiyama T., Mizuta K., Itoh Y. (2016). Effect of Polaprezinc on Oral Mucositis, Irradiation Period, and Time to Discharge in Patients with Head and Neck Cancer. Head Neck.

[22] Tsubura-Okubo M., Komiyama Y., Kamimura R., Sawatani Y., Arai H., Mitani K., Haruyama Y., Kobashi G., Ishihama H., Uchida D. (2021). Oral Management with Polaprezinc Solution Reduces Adverse Events in Haematopoietic Stem Cell Transplantation Patients. Int. J. Oral Maxillofac. Surg..

[23] Fujii H., Hirose C., Ishihara M., Iihara H., Imai H., Tanaka Y., Matsuhashi N., Takahashi T., Yamaguchi K., Yoshida K. (2018). Improvement of Dysgeusia by Polaprezinc, a Zinc-L-Carnosine, in Outpatients Receiving Cancer Chemotherapy. Anticancer Res..

